# Drug Interactions for Patients with Respiratory Diseases Receiving COVID-19 Emerged Treatments

**DOI:** 10.3390/ijerph182111711

**Published:** 2021-11-08

**Authors:** Marios Spanakis, Athina Patelarou, Evridiki Patelarou, Nikolaos Tzanakis

**Affiliations:** 1 Department of Nursing, School of Health Sciences, Hellenic Mediterranean University, GR-71004 Heraklion, Crete, Greece; apatelarou@hmu.gr (A.P.); epatelarou@hmu.gr (E.P.); 2 Computational BioMedicine Laboratory, Institute of Computer Science, Foundation for Research & Technology-Hellas (FORTH), GR-70013 Heraklion, Crete, Greece; 3 Department of Respiratory Medicine, University Hospital of Heraklion, Medical School, University of Crete, GR-71303 Heraklion, Crete, Greece; tzanakis@med.uoc.gr

**Keywords:** drug-drug interactions, COVID-19, ADRs, healthcare personnel, nursing personnel, respiratory diseases, COPD

## Abstract

Pandemic of coronavirus disease (COVID-19) is still pressing the healthcare systems worldwide. Thus far, the lack of available COVID-19-targeted treatments has led scientists to look through drug repositioning practices and exploitation of available scientific evidence for potential efficient drugs that may block biological pathways of SARS-CoV-2. Till today, several molecules have emerged as promising pharmacological agents, and more than a few medication protocols are applied during hospitalization. On the other hand, given the criticality of the disease, it is important for healthcare providers, especially those in COVID-19 clinics (i.e., nursing personnel and treating physicians), to recognize potential drug interactions that may lead to adverse drug reactions that may negatively impact the therapeutic outcome. In this review, focusing on patients with respiratory diseases (i.e., asthma or chronic obstructive pulmonary disease) that are treated also for COVID-19, we discuss possible drug interactions, their underlying pharmacological mechanisms, and possible clinical signs that healthcare providers in COVID-19 clinics may need to acknowledge as adverse drug reactions due to drug-drug interactions.

## 1. Introduction

Severe acute respiratory syndrome coronavirus 2 (SARS-CoV-2), the causative agent of the ongoing pandemic of coronavirus disease (COVID-19) is still pressing the healthcare systems worldwide to their limits, as the delta variant is now dominating. More than 250 million people have been infected, and the death toll, so far, from COVID-19 is over 5 million, while more than 100 million have recovered globally [1]. Since its outbreak in 2019 in China [2,3], there are tremendous efforts from the scientific community to develop efficient strategies in all aspects of healthcare, from preventive quarantine measures up to innovative approaches, such as precision medicine [4,5]. The lack of available targeted medicinal options and the spreading speed of the virus globally has stressed scientists to look for potential promising drugs through drug repositioning practices and exploitation of available scientific evidence to tackle the biological pathways that SARS-CoV-2 is using in the body [6,7]. Since the COVID-19 outbreak that led to the pandemic crisis and maybe for the first time in medicinal history, there are a great deal of available epidemiological studies and data to determine COVID-19 frequency measures globally. Thus far, it is well understood that the morbidity and mortality rates are higher in elderly people with underlying chronic diseases, such as cardiovascular diseases (CVD); diabetes; respiratory disorders, such as chronic obstructive pulmonary disease (COPD); diabetes; and cancer [8,9]. It is estimated that the overall fatality from COVID-19 in people with no underlying health problems is about 2–3%, while it is six- to ten-fold higher with people with one or more underlying health conditions. In this respect, given also that COVID-19 is referred to as an acute respiratory syndrome, it is of no surprise that underlying medical conditions that increase a person’s risk of severe illness from COVID-19 include respiratory disorders, such as COPD and asthma, cystic fibrosis etc. [10].

Till today, a number of medications have emerged as promising, and several medication protocols are followed, while several clinical trials are ongoing to enhance them and give a clear advantage in both the treatment of the virus and its pathological complications during patient hospitalization (Table 1) [11,12]. Most of the COVID-19-related hospitalizations refer to patients with underlying conditions that may need to receive additional medications for their chronic treatments [13]. One of the most important clinical considerations in cases of pharmacotherapy is the potential and clinically significant drug-drug interactions (DDIs) that may lead to unwanted adverse drug reactions (ADRs) with negative impact on patients’ health status [14].

DDIs describe the phenomenon when co-administered drugs share common molecular targets in biological pathways involved in their pharmacodynamics (PD) and pharmacokinetic (PK) processes. This results in a greater or lesser systemic exposure for one drug (victim) due to co-administration of another drug (perpetrator). DDIs are clinically significant when the modification in victim-drug exposure exceeds effective levels or reduced to sub-therapeutic concentrations depending on the perpetrator’s action as inhibitor or inducer for the shared biological pathway. Synergistic or competitive actions on the victim’s drug primary or secondary biological targets and signaling pathways are characterized as PD-DDIs (i.e., human ether-à-go-go-related gene, hERG, and QT-prolongation), while PK-DDIs occur when victim’s drug absorption, distribution, metabolism, and elimination (ADME) processes are altered [15]. Especially for PK-DDIs, the most often altered biological pathways are related with transport proteins (i.e., P-glycoprotein, P-gp; breast cancer resistance protein, BCRP; organic anion transporter polypeptides, OATPs; organic cation transporters, OCTs; multidrug and toxin extrusions, MATE; etc.), plasma proteins (i.e., albumin), and/or metabolic enzymes (i.e., Cytochrome P450, CYPs; Uridine 5’-diphospho-glucuronosyltransferases, UDPs; Glutathione S-transferases, GSTs; N-acetyltransferases, NATs; etc.) that define the PK parameters for a drug.

DDIs represent a major issue in clinical practice since potential modulation of the clinical outcome may result in adverse drug reactions (ADRs), a noxious and unintended response from a system organ (or whole body) to a drug, which occurs at doses normally used for the prophylaxis, diagnosis, or therapy of disease or for modifications of physiological functions [16,17,18]. DDIs can be evaluated and categorized in relation to their severity as of: (i) major importance due to adequate clinical observations that evaluates the combinations as “serious—avoid” and/or “contraindicated”; (ii) of “use with caution—monitor” or “moderate” importance, where clinical evidence suggest co-administration can be considered after a risk/benefit analysis; and (iii) of “moderate-minor” or “minor” significance, where DDIs may be described in experimental level but are not clinically observed [15,19].

In this review, we discuss potentially clinically significant DDIs of therapies administered in COVID-19 with an emphasis on drugs for respiratory disorders, their underlying pharmacological mechanisms, potential ADRs, and how healthcare personnel should be aware to recognize and manage them. Scientific information for COVID-19 candidate treatments in based on dashboard available from DrugBank or other relative sources [20]. The anatomic therapeutic classification (ATC) was used to extract classified drugs for respiratory disorders (ATC-R) that are used in chronic respiratory diseases. The drug interaction checkers from Medscape and Drugs.com was used to search available scientific evidence, along with the summaries of product characteristics (SmPC) or product information for all drugs. The characterization of the clinical significance for DDIs was based on available scientific evidence as described in previous works [21,22,23,24]. Briefly, assessment was based on various levels of evidence, such as in-silico/in-vitro/in-vivo data, clinical studies, expert opinion reviews, meta-analysis, and information encompassed in SmPCs. The clinical significance of the interactions in this study is represented as “Serious—Use alternative”, “Use with caution—Monitor” and “Moderate-Minor”.

## 2. COVID-19 Therapeutic Approaches and Drug Interactions

### 2.1. Emerged Drugs in COVID-19 Hospitalization and Their Interaction Potentials

Evidence for COVID-19 is advancing in an unprecedented speed for medical sciences. The lack of available medical options has led the scientific community to look through drug repositioning for potential drug candidates [25]. Following the evidence-based medicine hierarchy, a large volume of data starting from clinical observations up to observational studies, randomized clinical trials, and meta-analysis reports are continuously emerging [11,26]. Hence, COVID-19 Treatment Guidelines are continuously delivering relative direction to clinicians on how to provide healthcare for COVID-19 patients [1,27].

**Figure 1:**
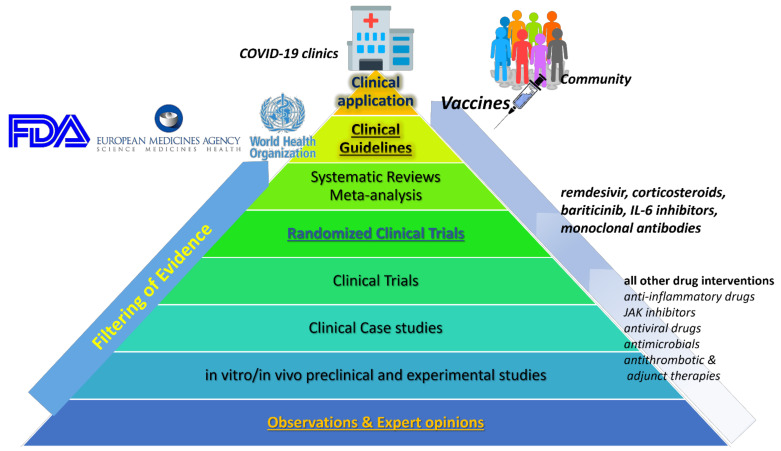
Classification of available medical options for COVID-19 emerged treatments based on available evidence hierarchy.

There are three primary pharmacological goals for treatment for COVID-19 hospitalized patients: (i) controlling of virus’s replication with antiviral drugs or drugs that modulate cellular mechanisms, which may play role in virus replication process (antimicrobials); (ii) supportive treatment for controlling of inflammation and immune response with glucocorticoids that are used in syndromes closely related to COVID-19 and/or with immunosuppressive agents, such as monoclonal antibodies; and (iii) reducing the risk for complications, such as thrombosis, with antiplatelet and aspirin as adjunct treatments [7,28,29,30]. Table 1 summarizes drugs that thus far have been emerged in COVID-19 [11], while their current level of evidence regarding their effectiveness on COVID-19 patients is depicted in Figure 1. Table 2 summarizes ADRs that may occur in COVID-19 patients and are related with DDIs, which are also discussed. More information with examples of drug combinations and the potential clinical outcome can be found in Supplementary File (Tables S1 and S2).

#### 2.1.1. Antiviral Medications in COVID-19 Patients and DDIs

Antiviral drugs have been thoroughly evaluated as medications that lower virus replication in COVID-19 but with debatable, inferior results from their use in terms of improved mortality rate. The exception is remdesivir, which was initially given an emergency use authorization (EUA) in May 2020 and, since October 2020, has received indication as a drug for treatment of COVID-19 disease in hospitalized adults or children >12 years and >40 kg [26,31]. Apart of their effectiveness, the studies focused on the observation of potential ADRs with one major risk factor to be capability related with PK-DDIs.

Atazanavir is a protease inhibitor and a direct-acting antiviral drug that has been tested for COVID-19 use [32]. Atazanavir is an inhibitor of CYP3A and UGT1A1, and potential co-administration with drugs that are substrates of CYP3A or UGT1A1 may result in PK-DDIs. Typical examples of serious-avoid DDIs are co-administration with other HIV-AIDs drugs, such as nucleoside reverse transcriptase inhibitors (NRTIs), such as lamivudine, etc.; non-nucleoside reverse transcriptase inhibitors (NNRTIS), such as efavirenz, etc.; anti-hepatitis C virus (anti-HCV) agents, such as grazoprevir; anticancer agents, such as irinotecan; immunosuppressants; antiarrhythmics (amiodarone, quinidine); Ca^2+^ channel blockers (bepridil, diltiazem, etc.); and lipid-lowering agents, such as statins. In all these cases, patients treated for COVID-19 with atazanavir may present ADRs due to prolonged pharmacological outcomes of the co-administered drugs from PK-DDIs and should be avoided or used with caution with continuous monitoring [33].

Darunavir is an HIV protease inhibitor suggested for COVID-19 although there is a lack of evidence for efficacy [34]. Darunavir is an inhibitor for CYP3A4, CYP2D6 and P-gp. The latter suggests that darunavir may lead to serious—avoid or use with caution—monitor PK-DDIs, with a wide number of medications, many of which have a narrow therapeutic index and are substrates for CYP isoenzymes and/or P-gp [35]. For example, co-administration with alfuzosin should be avoided since it may lead to hypotension. Co-administration with direct oral anticoagulants (DOACs), such as apixaban and rivaroxaban or platelet aggregation inhibitors (ticagrelor or clopidogrel), should be avoided, or dose adjustments and therapeutic drug monitoring (TDM) are needed to avoid any bleeding risk (or loss of action for clopidogrel). Similar precautions for co-administration should be made for warfarin although the active enantiomer (S-warfarin) is metabolized from CYP2C9 and only the R-warfarin from CYP3A4. In cases of anticonvulsants, especially those metabolized from CYP3A4, clinical monitoring is recommended. For patients administered antidepressants, such as Selective Serotonin Reuptake Inhibitors (SSRIs) or Tricyclic Antidepressants (TCAs), clinical monitoring and dose adjustments should be followed to avoid ADRs (nausea, dizziness, hypotension, and syncope). Similar precautions should be undertaken for antipsychotics that are metabolized from CYP3A4 or CYP2D6 (i.e., pimozide, quetiapine, risperidone) due to potential for serious and/or life-threatening ADRs, such as cardiac arrhythmias. In addition, sedatives, especially those metabolized from CYP3A, should not be co-administered to avoid prolonged sedation or respiratory depression. Co-administrations with voriconazole or other antifungals should be avoided or based on a risk-benefit analysis. The combination of darunavir with colchicine should be avoided, especially when renal and/or hepatic impairment exists due to potential of ADRs from the resulted PK-DDI, such as myopathy, neuropathy, multiorgan failure, and pancytopenia. Similar findings stand for cisapride due to the risk for cardiac arrhythmias. For patients with CVD receiving β-blockers, Ca^2+^ channel blockers, antiarrhythmics, or digoxin, use with caution—monitor is advised to avoid increased concentrations of CVD drugs, while ranolazine (metabolized from CYP2D6 and CYP3A4) and ivabradine (metabolized from CYP3A4) are a combination that should be avoided. In addition, due to the inhibition potential, darunavir should be avoided with statins metabolized from CYP3A4 due to the risk for rhabdomyolysis [33,36].

Lopinavir is a potent anti-HIV drug used in combination with ritonavir, considered a PK-DDI that boosts lopinavir’s pharmacological action through inhibition of hepatic-mediated metabolism of lopinavir from ritonavir. The combination is suggested to have some efficacy against COVID-19, but clinical data did not reveal any benefit [37,38]. However, the lopinavir/ritonavir combination is likely to increase plasma concentrations of drugs that are metabolized by CYP3A or are substrates of transporter proteins, such as P-gp, OATP1A2, OATP1B1, OATP1B3, OATP2B, and BCRP [39]. Co-administration with colchicine should be avoided due to life-threatening and fatal ADRs from DDIs (including rhabdomyolysis). Similar actions should be applied for CVD drugs, such as alfuzosin, ranolazine, and antiarrhythmics (i.e., amiodarone), while for digoxin, bepridil, Ca^2+^ channel blockers, and vasodilating agents (e.g., bosentan), caution and patient monitoring are suggested. The combination should be avoided also in cases of statins that are metabolized from CYP3A (lovastatin, simvastatin, etc.) due to the risk for rhabdomyolysis as well as for lomitapide. For patients with anticancer treatments caution, monitoring and dose adjustment to avoid treatment failure or ADRs from chemotherapy toxicity are suggested. Similarly for patients treated with anticoagulants, monitoring to avoid risk of bleeding should be followed. Lopinavir/ritonavir should be avoided for co-administration with central nervous system (CNS) drugs, such as antipsychotics, and should be used with caution and monitored when combined with benzodiazepines. For patients under antihistamine therapy with astemizole or terfenadine, the combination is contraindicated due to risk of serious arrhythmias. Finally, lopinavir/ritonavir should not be combined with tadalafil (CYP3A substrate) for patients with pulmonary arterial hypertension [40].

Favipavir is a pro-drug that activates through intracellular phosphorylation. Favipavir’s metabolism is mediated through aldehyde oxidase (AO) and xanthine oxidase (XO). According to SmPC, favipiravir is an irreversible inhibitor for AO in a dose- and time-dependent manner and an inhibitor of CYP2C8 in a dose-dependent manner. Pharmacogenomics of AO or XO should be considered for pharmacodynamic outcomes, and DDIs have yet to be determined clearly, including cases of drugs that modulate AO metabolism (e.g., cimetidine). Favipavir and its metabolite can inhibit organic-anion-transporters (OAT1, OAT3); thus, they may modulate the clearance of substrates, such as alcuronium and β-lactam antibiotics (cephalexin, flucloxacillin, penicillin, piperacillin, tazobactam) [41,42,43].

Remdesivir is a drug that has shown effectiveness against COVID-19 [31]. For remdesivir, the overall potential for interactions is currently unclear, and patients should be monitored although there is medium or low risk for DDIs [44,45]. In-vitro studies showed that remdesivir does not induce metabolic enzymes or transporters, while it has been described as a weak inhibitor for CYP3A, OATP1B1, OATP1B3, and MATE1. According to remdesivir’s SmPC, co-administration of medicinal products that are substrates of CYP3A4 or substrates of OATP 1B1/1B3 should be administered with a minimum 2-h gap between remdesivir administrations [46]. Attention is also needed for CYP3A4 inducers (i.e., barbiturates, rifampicin). To evaluate the potential for remdesivir for clinically significant DDIs, the use of physiologically based pharmacokinetic (PBPK) modeling and simulations (M&S) of clinical trials with virtual population groups was applied with Simcyp (v.18; Certara, Sheffield, UK). Simulations of co-administration with pravastatin, rosuvastatin, and midazolam showed a small AUC increase (5–10%), suggesting that remdesivir has a low potential to lead to PK-DDIs for patients treated for COVID-19 with usually administered doses. There are planned trials for PK evaluation of remdesivir in patients with hepatic and/or renal impairment (glomerular filtration rate, GFR < 30 mL/min). Till then, it is advised to use with caution and monitoring for co-administration of drugs that may increase the risk for hepatotoxicity or acute renal impairment [46].

Ribavirin was tested against COVID-19 although it was not associated with improved negative conversion time for SARS-CoV-2 test or with an improved mortality rate [47]. Ribavirin does not inhibit or induce CYP enzymes; thus, it is unlikely to be related with serious PK-DDIs. The combination with 6-mercaptopurine or azathioprine should be avoided due to increased risk for myelosuppression from inhibition of inosine monophosphate dehydrogenase by ribavirin that may lead to accumulation of metabolite 6-methylthioinosine monophosphate, which has myelotoxic action. The use of didanosine is contraindicated due to risk for fatal hepatic failure, pancreatitis, peripheral neuropathy, symptomatic hyperlactatemia, and lactic acidosis. Co-administration with drugs such as bexarotene may potentiate the risk of pancreatitis due to increased triglyceride levels. It may also decrease the hypoprothrombinemic effect of anticoagulants, such as warfarin/dicoumarol, and thus, INR monitoring is needed. Finally, patients with HIV-AIDs should be closely monitored when treated concomitantly with ribavirin and any of NRTIs to avoid any modulation in anti-HIV response [48].

#### 2.1.2. Antimicrobial Medications in COVID-19 and Drug Interactions

Azithromycin is a macrolide antibiotic used in patients with COVID-19 in combination with hydroxychloroquine to prevent risk of secondary infection and reduce mortality [49]. It is extensively distributed with high affinity in body tissues and especially in lungs. Azithromycin is a moderate-to-weak inhibitor of transporter proteins, such as P-gp, OATP1A2, and OATP2B1, and for CYP3A enzymes [50]. Due to P-gp inhibition, betrixaban should not be combined with azithromycin, while digoxin also appears to have elevated concentrations. Azithromycin should not be used with antacids, while nelfinavir and fluconazole may increase azithromycin’s concentrations with a PK-DDI of moderate significance, while a similar PK-DDI is considered to cause an increase in the Cmax for cyclosporin. The main issue of DDIs, though, for azithromycin is the QT-prolongation when it is used with antibiotics, such as rifabutin; antiarrhythmics, such as amiodarone; and 5-HT₄ receptor agonists, such as cisapride. Moreover, the combination itself with hydroxychloroquine may increase the risk for QT-prolongation in patients treated for COVID-19. Finally, prothrombin times should be monitored when azithromycin is co-administered with oral anticoagulants since there are some case reports for clotting formulation when combined with coumarin analogues and a theoretical risk for ergotism when combined with ergot derivatives [51].

Chloroquine and hydroxychloroquine are well known and caused a great speculation as possible effective drugs against COVID-19 [52]. Both drugs are known to increase the risk for QT-prolongation, and thus, their co-administration with drugs also triggering the same effect should be avoided or used with caution and monitored [53]. Hence, co-administration with antiarrhythmics (Class IA and III), tricyclic antidepressants, antipsychotics, antimicrobials (quinolones, antifungals, macrolides), and drugs for GI-mobility, such as cisapride, may lead to PD-DDIs and QT-prolongation that could be a serious ADR for COVID-19 patients. Antidiabetic drugs should be used with caution and under monitoring for avoidance of hypoglycemia, while with parasympathomimetics can lead to antagonism of action. Chloroquine and hydroxychloroquine can decrease the convulsion threshold and should be avoided or used with caution when anti-epileptics are co-administered. Regarding PK-DDIs, they can raise the serum digoxin levels as well as those of cyclosporine in transplanted patients and should be used with caution or under monitoring. Finally, co-administration with penicillamine should be avoided due to PK-DDIs that lead to hematological, renal, or skin reactions [54,55].

Ivermectin has been proposed to show antiviral activity for COVID-19 therapy [56]. Ivermectin is metabolized from CYP3A4 and is a substrate for P-gp. In addition, according to ivermectin’s SmPC, there are limited clinical data regarding potential clinically significant interactions with other drugs [57]. Hence, caution should be applied for possible dose adjustments when ivermectin is co-administered with potent P-gp and/or CYP3A4 inhibitors that may increase drug’s plasma concentrations.

Finally, nitazoxanide, although DDIs data are scarce, should be used with caution and monitor in cases of co-administration of drugs with high plasma protein binding and narrow therapeutic index, such as warfarin, valproic acid, and benzodiazepines [58,59].

#### 2.1.3. Immunomodulatory Medications in COVID-19 and Drug Interactions

Severe COVID-19 patients present a “cytokine storm” with cytokine release of interleukins, such as IL-1, IL-6, tumor necrosis factor (TNFa), and other inflammatory mediators, leading to a pulmonary inflammatory response making oxygenation challenging [60,61]. Drugs that are known to block inflammation processes related with hyper-inflammation due to the “cytokine storm” and respiration failure are continuously investigated. These drugs are monoclonal antibodies (mAbs) that act as interleukin inhibitors, drugs that target numb-associated kinase, corticosteroids, and convalescent plasma from donors that recovered from COVID-19.

The use of interleukin inhibitors, such as anakinra, canakinumab, infliximab, sarilumab, and tocilizumab, has been applied in COVID-19 patients to prevent severe respiratory failure [62,63]. Available evidence of DDIs between these mAbs with other medicines, such as non-steroidal anti-inflammatory drugs (NSAIDs), glucocorticoids, or antirheumatic drugs, is poor. Co-administration of other medications with potential immunosuppressive action, such as disease-modifying antirheumatic drugs (DMARDs) or chemotherapy, should be avoided due to the risk of immunosuppression and secondary infections [64,65,66]. Monitoring should be applied when patients are receiving medications that may lead to hepatotoxicity (i.e., efavirenz, methotrexate, etc.). Regarding PK-DDIs, inflammation process from elevated cytokine levels down-regulates the expression of hepatic CYP450 enzymes. Hence, as a secondary action, these mAbs may return to normal CYP450 abundance, which may “increase” (actually return to normal) the metabolic rates for substrate drugs [64,66,67,68]. This should be taken into consideration for COVID-19 patients that are continuing therapeutic administration of drugs that are substrates for CYP450 enzymes and with narrow therapeutic index and/or under therapeutic drug monitoring (i.e., warfarin, CNS drugs, such as alprazolam, etc.).

Bamlanivimab is an IgG1 mAb directed against the spike protein of SARS-CoV-2 and received an EUA by FDA in November 2020, which was recently revoked due to an increase in resistance to bamlanivimab SARS-CoV-2 viral variants, suggesting that potential benefits of bamlanivimab alone no longer outweigh the known and potential risks for the product [69]. Bamlanivimab does not show CYP450-mediated metabolism or renal metabolism; hence, PK-DDIs are unlikely.

Baricitinib is an inhibitor of Janus Kinase (JAK) interfering in intracellular signaling pathways of cytokines developed initially for rheumatoid arthritis. Regarding COVID-19, it received an EUA from the FDA based on evidence and clinical data showing that the combination with remdesivir was associated with reduction in recovery time and improvement of the overall clinical status for the patients [70]. According to baricitinib’s SmPC, administration should be avoided in patients taking strong inhibitors of organic anion transporters 3 (OAT3), such as probenecid [71,72]. Except that clear indication and despite that baricitinib seems to be a substrate for transporter proteins and partially metabolized from CYP3A4, sufficient evidence of potential DDIs is not available. Baricitinib has not been studied in combination with other JAK inhibitors or biologic DMARDs. However, patients receiving baricitinib with any of these drugs should be closely monitored for signs or symptoms of infection from immunosuppression during and/or after treatment. The same consideration should be followed to avoid any risk from PD-DDIs when myelosuppressive agents are administered. JAK inhibitors have been also associated with an increased risk of diverticulitis (DV) and gastrointestinal (GI) perforation in patients with risk factors or receiving treatments with agents associated with DV (i.e., aspirin) [71,72].

Colchicine is a pharmacological agent with anti-inflammatory action used in a variety of conditions (gout, pericarditis). Colchicine inhibits chemotaxis of neutrophils and inflammasome signaling and reduces the production of cytokines, which may be beneficial against inflammation-associated events in COVID-19; however, till today, data remain insufficient [73,74]. Regarding DDIs, colchicine is a substrate for P-gp as well as for CYP3A4/5. Hence, there is the risk for clinically significant PK-DDIs in patients receiving strong or moderate inhibitors for P-gp and/or CYP3A4/enzymes. Co-administration of cyclosporine with ranolazine, protease inhibitors, itraconazole, ketoconazole, fluconazole, erythromycin, and diltiazem should be avoided or used with caution to avoid toxic plasma levels and ADRs (e.g., diarrhea, nausea, cramping, abdominal pain, vomiting). Similarly, co-administration with inducers of CYP3A4 and/or P-gp could lead to sub-therapeutic effects for colchicine. Caution should also be given when it is co-administered with IL inhibitors mentioned earlier [75,76]. Renal failure can increase the risk for ADRs of colchicine, whereas macrolide antibiotics can induce pancytopenia, and cyclosporine can lead to neuromuscular related ADRs [77,78,79].

Fingolimod is an immunology modulator indicated as monotherapy for the treatment of patients with relapsing-remitting forms of multiple sclerosis (MS). Fingolimod has been tested for COVID-19 based on case reports regarding mild COVID-19 that mentioned continuation of the immunosuppressive drug with a full recovery without any complications, suggesting the need for further investigation regarding potential benefits from immunomodulation from fingolimod in COVID-19 patients [80]. Co-administration of fingolimod with anti-neoplastic or other immunosuppressive or immunomodulating agents should be avoided due to the enhanced risk for ADRs in immune system from PD-DDI synergisms. Patients receiving cardiovascular medications may present bradyarrhythmia following fingolimod first dose administration. Fingolimod has been associated with PR interval prolongation, QT-interval prolongation, and decreased heart rate. Thus, it should not be administered with antiarrhythmics, β-blockers, Ca^2+^ channel blockers, digoxin, and other drugs that decrease heart rate (e.g., cholinesterase inhibitors, pilocarpine, etc.). Regarding PK-DDIs, experimental data showed that fingolimod does not modulate abundance and/or the activity of CYP450 metabolic enzymes as well as hepatic uptake and/or efflux transport systems, so no clinically relevant interactions of substrates for these systems with the drug are expected at therapeutic concentrations. Co-administration of fingolimod with carbamazepine at maximal dose (600 mg) should be used with caution and monitor for potential sub-therapeutic effects [81,82].

Corticosteroids have anti-inflammatory, antifibrotic, and vasoconstrictive effects and have been evaluated in the past for their potential effectiveness in acute respiratory syndromes (ARDs), pneumonia, and septic shock. Corticosteroids have received scientific attention worldwide for their effectiveness against COVID-19. Thus far, available evidence supports an association between glucocorticoids (i.e., dexamethasone, methylprednisolone, hydrocortisone) and reduced mortality in COVID-19. Apart from their potential effectiveness against COVID-19, corticosteroids are well-known molecules with known DDIs. Special precautions should be made for potassium levels in blood (PD-DDIs with diuretics, laxatives, digoxin, etc.). For PK-DDIs, glucocorticoids can induce metabolizing enzymes, such as CYP3A11, CYP2B10, and mainly CYP3A4, due to its pharmacological action on nuclear pregnane X receptor (PXR). They also seem to induce transporter proteins, such as OATP2 and P-gp. The clinical significance of the resulted DDIs is dependent on the administered dosage and period of treatment [83,84,85,86].

Ruxolitinib is an antineoplastic agent administered in myelofibrosis and was investigated for potential effectiveness in COVID-19 against the “cytokine storm”. However, phase III study did not meet its primary endpoint or any relevant benefit among secondary and exploratory endpoints, including mortality and time to recovery [87,88,89,90,91]. Ruxolitinib is primarily metabolized by CYP3A4 and CYP2C9 enzymes with partial contribution of CYP1A2 and CYP2B6. Thus, all potential inhibitors for these enzymes may lead to clinically significant PK-DDIs and should be avoided or patients be monitored for potential ADRs from elevated concentrations of ruxolitinib. Regarding PD-DDIs, due to its pharmacological action to decrease heart rate and elevate PR, interval co-administration with antiarrhythmics, β-blockers, non-dihydropyridine calcium channel blockers, digitalis glycosides, cholinesterase inhibitors, sphingosine-1 phosphate receptor modulators, and HIV protease inhibitors should be avoided [92].

### 2.2. Drugs Administered in Respiratory Disorders and Potential DDIs in COVID-19 Patients

In this review, we focus on respiratory diseases, such as asthma, COPD, emphysema, cystic fibrosis, and bronchiectasis. These disorders are usually treated with systemic administration of drugs from ATC-R and mainly R03, drugs for obstructive airway diseases, through several biopharmaceutical products, such as inhalers, oral, or intravenous treatments. Table 3 summarizes the potential interactions and the ADRs that may be observed in COVID-19 patients and are related with DDIs when co-administered with treatment options for COVID-19. Additional information with examples of drug combinations of from ATC-R class with COVID-19 emerged treatments and the potential clinical outcome can be found in Supplementary File (Table S3).

#### 2.2.1. Selective β-2-Adrenoreceptor Agonists (R03AC) and COVID-19 Treatments

Selective β2-adrenoreceptor agonists (β2-agonists, ATC-R03AC) salbutamol, terbutaline, etc., can alter their pharmacological profile when co-administered with azithromycin, chloroquine, or hydroxychloroquine and the underlying moderate risk for QT prolongation from PD synergism [93]. For salmeterol and indacaterol, there is also the potential of PK-DDIs in cases that atazanavir, ritonavir, and/or darunavir are used [94,95,96]. Lastly, the co-administration of β2-agonists with corticosteroids should be given as beneficial in asthma or COPD considering the minor chance of hypokalemia [97].

#### 2.2.2. Glucocorticoids (R03BA) and COVID-19 Medications

Glucocorticoids are a drug category that is used in acute therapy for severe COVID-19. However, the co-administration with other COVI-19 treatments should be provided with considerations for potential DDIs for some antiviral agents or immunosuppressants. Atazanavir and ritonavir should be avoided or used with caution with glucocorticoids due to CYP3A4 and P-gp inhibition [86,96]. Anakinra, baricitinib, and canakinumab, when co-administered, can induce neutropenia due to PD-DDI between glucocorticoids and other immunosuppressants [64,98]. Combination of darunavir with corticosteroids should be avoided because either the inhibition of CYP3A4 increase the risk for Cushing’s syndrome or the induction of CYP3A4 from corticosteroids may reduce the concentration of darunavir [35,86].

#### 2.2.3. Anticholinergics (R03BB) and COVID-19 Treatments

There are not any known interacting mechanisms when anticholinergics, such as ipratropium and oxitropium, are co-administered with COVID-19 treatments. The only exception is revefenacin, which is a long-acting muscarinic antagonist used as a bronchodilator in COPD. Revefenacin should be used with caution and monitored in cases of co-administration with atazanavir, ritonavir, and nitazoxanide due to their capability to inhibit OAT1B1/1B3 [99].

#### 2.2.4. Xanthines (R03DA) and COVID-19 Treatments

Co-administration of xanthines (aminophylline, theophylline.) can lead to PK-interactions with interleukin inhibitors that modulate the abundance of various hepatic microsomal enzymes, including CYP1A2, 2C9, 3A4, etc. [64,65,66]. Similarly, co-administration with macrolide antibiotics, such as azithromycin, may alter the levels of xanthines (e.g., theophylline) due to CYP inhibition (CYP3A4). Finally, ritonavir may induce CYP1A2, decreasing the plasma concentrations and possibly diminishing the therapeutic response to theophylline; thus, dosage adjustment maybe required [40,100].

#### 2.2.5. Leukotriene Receptor Antagonists (R03DC) and COVID-19 Treatments

The orally administered leukotriene receptor antagonists (LTRA) are used for the chronic treatment of asthma. There are potential DDIs between LTRAs and COVID-19 treatments related mostly with zafirlukast and antivirals, chloroquine, hydroxychloroquine, or immunosuppressants, such as colchicine or tocilizumab, that could lead to PD-DDI-related neutropenia [101,102,103]. Additionally, there is a risk for hepatotoxicity when remdesivir is co-administered but of moderate-minor importance, and the same goes for zileuton [44,104]. A PK-DDI due to CYP3A4 inhibition from atazanavir and ritonavir can occur, but it is of minor significance. According to zafirlukast’s SmPC, there is a moderate PK interaction with aspirin with no clear biological mechanism and of moderate significance that may require dose adjustments for zafirlukast due to elevated concentrations (up to 45%) [105]. Finally, for montelukast, clinical monitoring due to moderate risk for reduced therapeutic effects may be appropriate when CYP-inducers, such as dexamethasone, etc., are used [106,107].

#### 2.2.6. Antihistamines (R06) and COVID-19 Treatments

DDIs may occur when astemizole or terfenadine are co-administered with antiviral drugs that inhibit CYP3A4, such as atazanavir, azithromycin, and ritonavir [96]. Ritonavir can also modulate the renal elimination of cetirizine or levocetirizine and the CYP2D6-mediated metabolism of loratadine [40]. These combinations should be used with caution and monitored for the avoidance of any potential ADRs. Astemizole or terfenadine could also contribute to QT-prolongation due to PD-DDI with azithromycin, chloroquine, or hydroxychloroquine.

## 3. Healthcare Personnel in COVID-19 Clinics: Identifying and Manage DDIs and ADRs

SARS-CoV-2 is here to stay, and although the available state-of-the-art vaccines will keep on eliciting and hopefully smoothing the pandemic waves, viral lineages will continue to emerge and circulate globally, challenging the healthcare systems [108,109,110]. Vaccines have proven beyond doubt their efficacy as preventive interventions against pandemic waves, while recently it was announced that the benefit of vaccination programs in the U.S. can only be associated with a reduction of about 140 thousand less deaths from COVID-19 and a statistical life benefit of approximately $1 trillion [111,112]. Even though vaccines are the best “weapon” to date for pandemic cessation, prevention will never be enough, and the need to develop advanced therapeutic options for COVID-19 or potential other appearing viruses is beyond doubt [113,114,115]. Among other issues, drug repurposing procedures can be a solution, and although in the case of SARS-CoV-2, the data till today are not encouraging, the “research battle” rages on [12,115,116]. Utilizing the power of digital evolution and of systems pharmacology tools, state-of-the-art artificial intelligence (AI), and machine learning (ML) approaches have been widely applied for drug screening and repurposing to find possible breakthroughs in drug research and development (R&D) for novel therapies or drug repositioning for SARS-CoV-2 [116,117,118,119,120].

Recently, as this review is submitted, there was the announcement for molnupiravir, a nucleoside analogue that interferes within SARS-CoV-2’s RNA replication for which clinical trials of phase 3 are coming to an end, and the drug will seek emergency authorization license for patients at mild-to-moderate or high risk of developing severe COVID-19 [121]. Whatever the case may be, SARS-CoV-2 challenges scientists in academia and industry “benches”, while at the same time, it puts to test the “bedside” of healthcare systems [114]. Looking at it from a mirror perspective for comorbidities in COVID-19 patients, it is of no exaggeration to state that for the first time in the modern healthcare era, COVID-19 is the common underlying disease for so many different clinical cases of patients with comorbidities and so many different clinical scenarios [13,122].

The total distribution and determinants of health-related states or events in COVID-19 patients is an ongoing perspective unceasingly evaluating towards population-based health management frameworks and clinical protocols. One of its main aspects is the need for optimal healthcare provision and the minimization of any disease and/or drug-related adverse events. DDIs either of PK and/or PD mechanisms represent a crucial clinical aspect in healthcare provision and a key factor associated with ADRs, which not only prolong the hospitalization but also jeopardize the patient’s health status and response to treatment. This medical fact, of course, has not gone unnoticed for drugs used in COVID-19 patients [123,124,125]. The awareness and management of DDIs among healthcare personnel not only enhances treatment outcomes but also improves the healthcare provision, hospitalization periods, and overall healthcare costs. This is especially important in the case of the COVID-19 pandemic and the unprecedented pressure that was put on hospitals regarding their human resources and/or Intensive Care units (ICU) equipment, which during the COVID-19 crisis put to the test. As Dr. Arabi et al. recently and in a sufficient way stated, COVID-19 will bring transformative changes in how critical care is provided, and it demonstrates the demands for upgraded ICU bed capacity, flexible ICU staffing, reliable supply chains for personnel, protective equipment, ICU devices, consumables, pharmaceuticals, establishment of ICU triage principles, improved communication with families, digital transformation, and more agile, collaborative research [126].

The other way that COVID-19 challenges the healthcare eco-system refers to the awareness of healthcare teams to exploit their knowledge regarding optimum healthcare provision in complicate clinical scenarios [127]. Since the pandemic outbreak, clinical protocols were established following exploitation of pharmacological mechanisms for several drugs, drug-repurposing, and evidence-based approaches to find effective therapies for hospitalized patients. This universality of the disease creates different and complicated clinical scenarios especially for infected patients with comorbidities and complex therapeutic schemes. Especially for chronic diseases for which patients seem to follow a stable treatment that allows them to manage their condition (i.e., COPD, asthma, or other respiratory diseases), the complications of COVID-19 may raise new challenges and needs for therapy adjustments. Similar tasks may be raised for other cases of patients that receive chronic treatments, such as people with epilepsy or people with coagulation problems, to name some of a few [54,55,128,129,130,131]. Therefore, it is crucial that healthcare professionals, especially those in COVID-19 clinics, be as aware as possible regarding responsiveness and management of adverse drug events (ADEs), ADRS, and/or DDIs in order to ensure drug safety and avoid any misdiagnosis [94,95,123,124,125,132,133]. Among healthcare professionals, clinical pharmacists as members of multidisciplinary teams can contribute significantly to the prevention, reduction, and management of drug-related problems, such as DDIs and ADRs, during patient hospitalization [134,135,136]. The pivoting role of clinical pharmacists not only can be overlooked but already, available data show their contribution in evaluating clinical scenarios for symptom assessment (including ADRs) and treatment decisions for optimized therapeutic schemes as well as patient education during hospitalization in COVID-19 recovery clinics [137,138]. Especially for COVID-19 clinics, recent results from available studies focusing on the role of clinical pharmacists on COVID-19 hospitalized patients further support their effective role in healthcare units on drug-related problems and especially recognition, prevention, and management of ADRs and DDIs [139,140,141,142].

**Figure 2:**
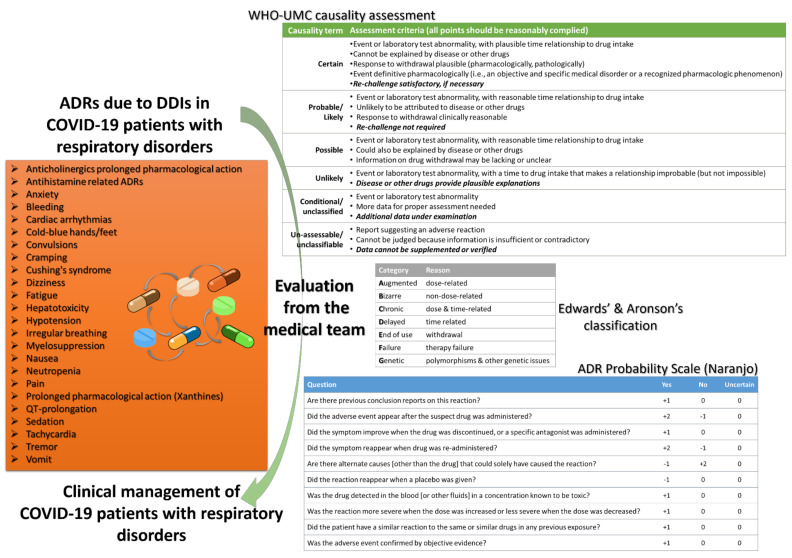
Potential ADRs for COVID-19 patients with respiratory disorders and evaluation tools regarding causality assessment (Naranjo scale interpretation of scores: ≥9 definite, 5–8 probable, 1–4 possible, ≤0 doubtful).

There are many ways to approach an ADR event, such as Naranjo ADR probability scale, other mnemonic scales, or causality categories, such as the Uppsala’s Monitoring Centre in collaboration with WHO (WHO-UMC) or Edward’s and Aronson’s categorization [18,143,144]. Indifferent from the evaluation scale that is used, the medical teams in COVID-19 clinics (doctors, nurses, clinical pharmacists, supporting staff, etc.) should be aware and capable to evaluate and manage the potential ADRs from DDIs and distinguish them from COVID-19 symptoms, especially those related with the cardiovascular, central nervous, respiratory, and gastrointestinal system (Figure 2). Extending this reasoning, COVID-19 also showed the need for fusing evidence-based medicine knowledge with precision medicine and personalized healthcare approaches to address all the potential clinical scenarios.

In this review, we presented repurposed drugs for COVID-19 patients, and we discussed their capability to present DDIs with a special focus on patients with respiratory disorders. We excluded the case of lung cancer pharmacotherapy since these patients by default are a different category that require specific clinical protocols and supportive care [10,123]. Advancing previous works, we discussed also potential clinical signs that could be strongly related with ADRs from occurring DDIs and not with disease progression, and it is crucial for healthcare professionals to be capable to distinguish between them as part of their diagnosis procedure [142,143,144]. Overall, one of the most often occurring ADRs is QT prolongation, which can be a result of an occurring DDI also. However, despite that being the most prevalent, cardiac arrhythmias are not the only occurring ADRs that healthcare professionals should be aware of (Figure 2).

Antivirals that are administered on the clinical level to COVID-19 patients are known to lead to PK-DDIs regarding inhibition of transporter proteins and metabolic enzymes, thus leading to prolonged pharmacological action for co-administered medications that may be clinically expressed as ADRs and toxicity. This can be related with typical and manageable clinically symptoms for COVID-19 patients, such as nausea, vomiting, fatigue, and constipation. However, till today, for the proposed COVID-19 medicines, there are cases that are more complex ADRs from DDIs, such as irregular breathing, bleeding, hypotension, sedation, and arrhythmias, that may require additional clinical experience to distinguish them as ADRs associated with DDIs and not with COVID-19 pathophysiology and progression. For this reason, the co-administration of the proposed COVID-19 medications should be made with considerations to modulate or substitute drugs that the patient is administered for comorbidities, or at least administer them under clinical monitoring to avoid any ADRs (Table 2).

Regarding respiratory diseases, such as asthma, COPD, emphysema, cystic fibrosis, and bronchiectasis, the presented DDIs for COVID-19 patients were related with ADRs that can be of “use with caution—monitor” or “moderate-minor” clinical significance (Table 3). Patients treated with β2-agonists may develop arrhythmias due to QT prolongation or other relative cardiovascular incidents, such as tachycardia, with several COVID-19 emerged treatments. The co-administration of immunosuppressants in patients receiving glucocorticoids should take into consideration the risk for neutropenia and, if darunavir is co-administered, the risk for Cushing’s syndrome. The inhibition ability against drug-metabolizing enzymes for antivirals can modulate the PK properties for some anticholinergics, such as revefenacin, and prolong its pharmacological action. The use of interleukin inhibitors to sustain the “cytokine storm” and reduce inflammation should always consider their impact on drug metabolic enzymes and transport proteins. Neutropenia may also be observed for patients prescribed LTRAs and administered antivirals or immunosuppressants for COVID-19, whereas if remdesivir is administered, there is a risk for hepatotoxicity. Finally, for antihistamines, there is a moderate risk for prolonged pharmacological action and ADRs when antivirals are administered, and caution should be followed when antimicrobials are co-administered with antihistamines to avoid any risk for QT prolongation. Thus, it is important that if any of these ADRs occur in a hospitalized COVID-19 patients with respiratory diseases, the medical team should be able to evaluate them and adjust the therapeutic protocol as needed (Figure 2), such as dose adjustments with reducing the administered dose or modulating time intervals and therapeutic drug monitoring of necessary biomarkers [145,146,147,148].

**Figure 3:**
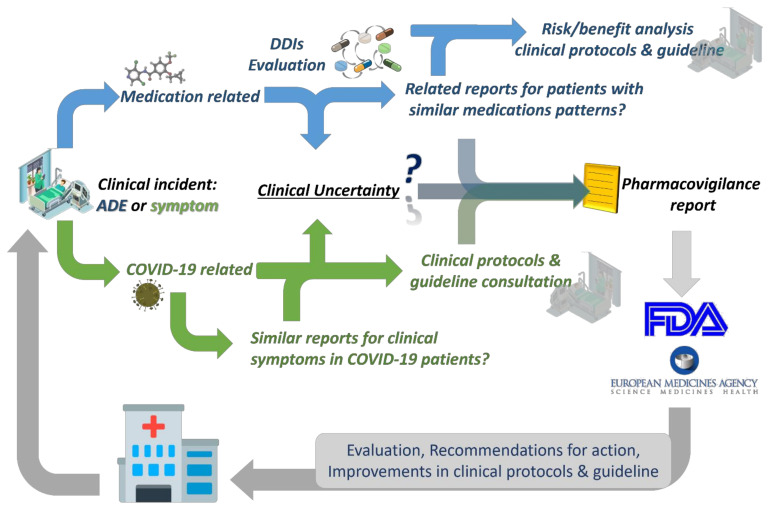
The importance of appropriate management of symptoms or ADEs for a COVID-19 patient and how healthcare professionals can actively participate in the analysis of different clinical scenarios, so regulatory can provide relative feedbacks improving clinical protocols.

The management of ADRs and DDIs from healthcare personnel, especially doctors, clinical pharmacists, and nurses who work in COVID-19 and ICU clinics, is crucial for optimal healthcare provision. Recently, it was estimated that ~40% of COVID-19 patients are exposed to at least one clinically significant DDI with antiviral drugs, with protease inhibitors as the most often associated medications in DDIs, whereas the number of patients in other works reaches up to 60% [94,134,149]. This shows that COVID-19 patients are of high risk for DDIs, as expected, and healthcare professionals should be aware of their management [14]. As the scientific community is still “educating” itself regarding all the biological background of SARs-CoV-2, it is of high importance the pharmacovigilance procedures for the optimum healthcare management. It is crucial that any deviation from the clinical protocols for a patient diagnosed with COVID-19 should be evaluated and reported so to be further analyzed from the respective stakeholders (regulatory, authorities, etc.). Thus, all the gathered knowledge can be filtered through appropriate channels from pharmacovigilance regulatory bodies to be evaluated, revised, and return to enrich the clinical protocols (Figure 3). Such an active “pharmacovigilant” role of healthcare professionals will boost also public confidence in their healthcare eco-systems against misinformation and same time will advance their role in the new era of healthcare [150,151,152,153].

## 4. Conclusions

COVID-19 is here to stay and will continue to challenge healthcare systems worldwide. COVID-19 does not discriminate health status, and it is especially harsh for patient population groups with comorbidities, such as patients with additional respiratory disorders. Apart of the vaccination programs that try to restrain the speed of the pandemic waves through development of herd immunity and reduce the risk for severe acute respiratory syndrome, it is important to develop potential treatments for the those that will be eventually infected and need hospitalization. Thus, one of the key issues is the awareness of healthcare professionals for optimization of healthcare provision and minimization of any ADEs, especially ADRs causally related with DDIs. Although DDIs for patients with respiratory disorders are mainly of moderate importance, some cases exist in which DDIs could be related with clinically significant outcomes, and special precautions should be considered. Thus far, treatment goals for hospitalized COVID-19 patients are to control inflammation and immune response, prevent complications from the disease progression, and avoid the acute respiratory distress syndrome (ARDS). In order for this goal to be achieved, a stepwise approach should be followed, including prediction and avoidance of DDIs and ADRs and proper healthcare. This requires a medical team of physicians, clinical pharmacists, and nurses with high awareness of effective management of drug-related problems and capability of risk-benefit analysis of patients’ health status. Tools such as clinical decision support systems can assist in prioritization of actions, including optimum treatment options, for patients in high risk, such as those with respiratory disorders comorbidities, whereas drug administration should be based on the AVOID Mistakes mnemonic code (Allergies, Vitamins or other supplements, Over-the-counter-drugs, Interactions, Disease, and Mendel) for COVID-19 patients that are first-time admitted in the clinic. This review focused on DDIs of COVID-19 treatment strategies with respiratory medicines. Assured conclusions about which of those scenarios should require clinical considerations in clinical settings will be revealed after systematic analysis of undergoing clinical studies that examine different clinical groups of COVID-19 patients for DDIs.

## Figures and Tables

**Table 1:** Emerged drugs administered in COVID-19 patients.

Antivirals	Antimicrobials	Immunomodulators	Adjunct Agents
Atazanavir	Azithromycin	Anakinra	Aspirin
Darunavir	Chloroquine	Bamlanivimab	Bevacizumab
Fabifavir	Hydroxychloroquine	Baricitinib	Dalteparin
Lopinavir/Ritonavir	Ivermectin	Canakinumab	Enoxaparin
Remdesivir	Nitazoxanide	Colchicine	
Ribavirin		Dexamethasone	
		Fingolimod	
		Hydrocortisone	
		Methylprednisolone	
		Ruxolitinib	
		Sarilumab	
		Tocilizumab	

**Table 2:** Potential DDIs in COVID-19 medications, their clinical significance, and clinical signs of ADRs related with them. (PK, pharmacokinetics, i.e., transport and metabolism processes; PD, pharmacodynamic, i.e., potassium Ccannels in heart, convulsion thresholds).

COVID-19 Medications	Co-Medications	DDI Type	Significance	Clinical Signs-ADRs
Antivirals	HIV-AIDs	PK	Serious—Avoid or Monitor	Fatigue, irregular breathing, cold-blue hands/feet, nausea
	Antineoplastic	PK	Serious—Avoid or Monitor	Tiredness, nausea, vomiting, anemia, etc.
	Immunosuppressants,	PK	Serious—Avoid or Monitor	Secondary infections, nausea, vomiting, tremors, etc.
	Antiarrhythmics/Ca^2+^ blockers	PK	Serious—Avoid or Monitor	Hypotension, edema, constipation, drowsiness, nausea, rash
	DOACs/anticoagulants	PK	Use with caution	Bleeding risk, bleeding signs
	SSRIs or TCAs	PK	Use with caution	Nausea, dizziness, hypotension, syncope
	Antipsychotics	PK	Serious—Avoid or Monitor	Cardiac arrhythmias
	Sedatives	PK	Serious—Avoid or Monitor	Prolonged sedation
	GI-track	PK	Serious—Avoid or Monitor	Cardiac arrhythmias
	Antihistamines	PK	Use with caution	Cardiac arrhythmias
Antimicrobial	Anti-infectives (antibiotics, antifungals, antimalarials)	PD	Serious—Avoid or Monitor	QT-prolongation
	GI-agents	PD	Serious—Avoid or Monitor	QT-prolongation
	Psychotropic (antipsychotics, SSRIs, TCAs)	PD	Serious—Avoid or Monitor	QT-prolongation
	Analgesics (opioids)	PD	Serious—Avoid or Monitor	QT-prolongation
	Antihistamines	PD	Serious—Avoid or Monitor	QT-prolongation
	Antiarrhythmics	PD	Serious—Avoid or Monitor	QT-prolongation
	Anesthetics	PD	Serious—Avoid or Monitor	QT-prolongation
	Antiepileptics (with chloroquine)	PD	Use with caution	Convulsions
Immunomodulatory	DMARDs	PD	Serious—avoid or monitoring	prolonged pharmacological action, myelosuppression toxicity
	TDM, narrow therapeutic index, increased first-pass effect drugs	PK	Use with caution	Increased metabolism modulation of steady-state and drug response
	Colchicine-CYP inhibitors	PK	Use with caution	Nausea, dizziness, cramping, pain, vomit

DDI: drug-drug interactions; ADRs: adverse drug reactions; DOACs: direct oral anticoagulants; SSRIs: Selective serotonin reuptake inhibitors; TCAs: tricyclic antidepressants; DMARD: disease-modifying antirheumatic drugs; TDM: therapeutic drug monitoring; CYP: Cytochrome P-450.

**Table 3:** DDIs mechanism and significance between drugs used in respiratory disorders and COVID-19 medications along with potential ADRs that may occur in those patients.

Respiratory Medications	COVID-19 Medications	DDI Type	Significance	Clinical Signs-ADRs
β2-agonists	chloroquine/hydroxychloroquine	PD	Use with caution	QT prolongation
	antivirals	PK	Moderate	prolonged pharmacological action-tachycardia, anxiety, tremor
Glucocorticoids	immunosuppressants (interleukin inhibitors)	PD	Moderate	Neutropenia
	darunavir	PK	Use with caution	Cushing’s syndrome
Anticholinergics	antivirals (lopinavir/ritonavir)	PK	Use with caution	prolonged pharmacological action
Xanthines	immunosuppressants (interleukin inhibitors), antivirals	PK	Use with caution	prolonged pharmacological action
LTRA	antivirals, chloroquine, hydroxychloroquine, immunosuppressants	PD	Use with caution	Neutropenia
	remdesivir	PD	Moderate	hepatotoxicity
Antihistamine	antivirals	PK	Moderate	prolonged pharmacological actionantihistamine related ADRs
	Antimicrobials (azithromycin, cholorquine, etc.)	PD	Use with caution	QT prolongation

## Data Availability

Publicly available datasets were utilized in this study along with information from product monographs and summaries of product characteristics as they are referenced. Online resources: Drugbank (www.drugbank.com); WHO-ATC/DDD Index 2021 (https://www.whocc.no/atc_ddd_index/) Medscape (https://reference.medscape.com/drug-interactionchecker); Drugs.com (https://www.drugs.com/interaction/list/).

## Supplementary Materials

The following are available online at https://www.mdpi.com/article/10.3390/ijerph182111711/s1, Table S1: Substrates, inducers and inhibitors for transport proteins, metabolic enzymes and plasma proteins for drugs that are discussed in this work; Table S2: Drug-Drug interactions between emerged treatments for COVID-19 and other medications; Table S3: Drug-Drug interactions between emerged treatments for COVID-19 and respiratory medications.
