# Supplementary material for: Drug Interactions for Patients with Respiratory Diseases Receiving COVID-19 Emerged Treatments
Source: Int J Environ Res Public Health. 2021 Nov 8;18(21):11711. doi: 10.3390/ijerph182111711 (PMC8583457; doi:10.3390/ijerph182111711)
Supplement: Supplementary file 1 [file ijerph-18-11711-s001.zip › ijerph-1399561-supplementary.pdf]

Supplementary Material: Drug interactions for patients with respiratory diseases receiving COVID-19 emerged treatments

Marios Spanakis, Athina Patelarou, Evridiki Patelarou and Nikolaos Tzanakis

The supplementary file contains additional information as supplementary tables regarding the scientific data that were presented in the manuscript regarding detailed cases of PK and/or PD interactions that could lead in occurrence of ADRs for COVID-19 patients.

Table S1. Substrates, inducers and inhibitors for transport proteins, metabolic enzymes and plasma proteins for drugs that are discussed in this work. (X: inhibitor, S: substrate, I: inducer)

	Transport proteins				***Metabolizing enzymes***				***Plasma proteins***
Drugs for COVID-19	**P-gp**	**OATP/ OCTP**	**MATE**	**BCRP**	**CYP3A**	**CYP2D6**	**Other CYPs or other enzymes**	**UGT**	**albumin**
Antivirals									
Darunavir	X				X	X			
Fabifavir		X (OAT1 & OAT3)					X (CYP2C8, AO, XO)		
Lopinavir/ Ritonavir	X	X OATP1A2, OATP1B1, OATP1B3, OATP2B1)		X	X				
Remdesivir		X (OATP 1B1/1B3)	X		X				
Antimicrobials									
Azithromycin	X	X OATP1A2 OATP2B1			X			X UGT1A1	
Ivermectin	S				S				
Nitazoxanide									S
Immunomodulators									
Anakinra, Canakinumab Sarilumab Tocilizumab							Increasing enzymatic abundance		
Baricitinib	S	S			S				
Colchicine	S				S				
Dexamethasone									
Hydrocortisone	I	I (OATP2)			I (+CYP3A11)				
Methylprednisolone									
Ruxolitinib					S		S (CYP1A2, CYP2C9, CYP2B6)		
Medications for respiratory diseases									
Xanthines					S		S (CYP1A2, CYP2C9,)		
zafirlukast					S				
Montelukast					S				
Astemizole					S				
Cetirizine/levocetirizine						S			

Table S2. Drug-Drug interactions between emerged treatments for COVID-19 and other medications (Clinical Significance: Serious–Use alternative= +++, Use with caution-Monitor= ++, moderate-minor=+)

Drugs in COVID-19		DDI	Biological Pathways involved/ Clinical result	Clinical significance	Examples of drugs that can be result in DDIs
**Antivirals**	Atazanavir	PK	CYP3A4, UGT1A1 (inhibitor)	+++	NRTIs, NNRTIS, anti-HCV, irinotecan, amiodarone, quinidine, diltiazem, bepridil, statins
	Darunavir	PK	CYP3A4, CYP2D6, P-gp (inhibitor)	+++	alfuzosin, DOACs, PAIs, anticoagulants, anticonvulsants SSRIs, TCAs, antipsychotics, voriconazole, colhicine, cisapride, β-blockers, Ca2+blockers, ranolazine, ivabradine, statins
	Favipavir	PK	AO, CYP2C8	+	amodiaquine, cerivastatin, dasabuvir, enzalutamide, imatinib, loperamide, montelukast, paclitaxel, pioglitazone, repaglinide, and rosiglitazone
	Lopinavir/ ritonavir	PK	CYP3A4, OATP1A2, OATP1B1, OATP1B3, OATP2B1, BCRP	+++	colchicine, alfuzosin, ranolazine, antiarrhythmics, statins, lomitapide, anticoagulants, astemizole, terfenadine, tadalafil
	Remdesivir	PK	CYP3A, OATP1B1, OATP1B3, MATE1	++	Dexamethasone, rifampicin, barbiturates, ACE inhibitors, antiviral agents, beta-lactam antibiotics, antineoplastics, metformin, NSAIDs, antiandrogens, barbiturates, statins, midazolam
	Ribavirin	PD	myelosuppression, hepatoxicity, pancreatitis hypoprothrombinemic, anti-HIV	+++	6-mercaptopurine, azathioprine, didanosine, bexarotene, anticoagulants, NRTIs
**Antimicrobial**	Azithromycin	PK	P-gp, OATP1A2, OATP2B1, CYP3A	++	betrixaban, digoxin, antacids, nelfinavir, fluconazole, cyclosporin
	Azithromycin, Chloroquine, Hydroxychloroquine	PD	hERG -> QT-prolongation	+++	amifampridine; amiodarone; amisulpride; amitryptiline; apomorphine; bedaquiline; bosutinib; cabozantinib; ceritinib; chlorpromazine; ciprofloxacin; cisapride; citalopram; clarithromycin; clomipramine; crizotinib; dasatinib; delamanid; deofetilide; desipramine; disopyramide; dolasetron; doxepin; dronedarone; droperidol; efavirenz ;encorafenib; eribulin; erythromycin; escitalopram; flecainide; fluconazole; fluoxetine; haloperidol; hydroxyzine; ibutilide; imipramine; inotuzumab; itraconazol; eketoconazol; elapatinib; lenvatinib; levofloxacin; levomepromazine; lithium; lofexidine; methadone; moxifloxacin; nilotinib; ondansetron; paliperidone; pazopanib; pimozide; procainamide; quetiapine; quinine; ranolazine; ribociclib; risperidone; saquinavir; sertraline; sildenafil; sorafenib; sotalol; sulpiride; sumatriptan; sunitinib; tetrabenazine; thioridazine; tizanidine; tolterodine; toremifene; vandetanib; vardenafil; vemurafenib; venlafaxine; voriconazole; ziprasidone; zolmitriptan
		PD	Convulsion threshold	+++	antiepileptics
		PD	Glucose homeostasis	++	Diabetes I drugs
		PD	ANS	+	parasympathomimetics
	Ivermectin	PK	P-gp, CYP3A	++	clarithromycin, erythromycin, diltiazem, ranolazine, itraconazole, ketoconazole, ritonavir, verapamil, grapefruit
	Nitazoxanide	PK	Protein binding affinity	++	benzodiazepines, warfarin, valproic acid

**Immunomodulators**	Anakinra,<br>Canakinumab<br>Sarilumab<br>Tocilizumab	PK	CYP abundance	++	CYP substrates, especially drugs of narrow therapeutic index atorvastatin, calcium channel blockers, theophylline, warfarin, phenytoin, ciclosporin, benzodiazepines etc.
	Baricitinib	PK	OAT3	+++	probenecid
		PD	immunosuppression	+++	JAK inhibitors or other biologic DMARDs
	Colchicine	PK	P-gp, CYP3A (substrate)	++	clarithromycin, erythromycin, diltiazem, ranolazine, itraconazole, ketoconazole, ritonavir, verapamil, grapefruit
	Fingolimod	PD	immunosuppression	+++	immunomodulating agents
			hERG -> QT-prolongation	+++	amifampridine; amiodarone; amisulpride; amitryptiline; apomorphine; bedaquiline; bosutinib; cabozantinib; ceritinib; chlorpromazine; ciprofloxacin; cisapride; citalopram; clarithromycin; clomipramine; crizotinib; dasatinib; delamanid; deofetilide; desipramine; disopyramide; dolasetron; doxepin; dronedarone; droperidol; efavirenz ;encorafenib; eribulin; erythromycin; escitalopram; flecainide; fluconazole; fluoxetine; haloperidol; hydroxyzine; ibutilide; imipramine; inotuzumab; itraconazol; eketoconazol; elapatinib; lenvatinib; levofloxacin; levomepromazine; lithium; lofexidine; methadone; moxifloxacin; nilotinib; ondansetron; paliperidone; pazopanib; pimozide; procainamide; quetiapine; quinine; ranolazine; ribociclib; risperidone; saquinavir; sertraline; sildenafil; sorafenib; sotalol; sulpiride; sumatriptan; sunitinib; tetrabenazine; thioridazine; tizanidine; tolterodine; toremifene; vandetanib; vardenafil; vemurafenib; venlafaxine; voriconazole; ziprasidone; zolmitriptan
			bradyarrhythmia	++	Cardiovascular medications
	Dexamethasone<br>Hydrocortisone,<br>methylprednisolone	PD	potassium homeostasis	++	diuretics, laxatives, digoxin
		PK	P-gp, OATP2, CYP3A11, CYP2B10, CYP3A4 (inducers)	+	CYP substrates, especially drugs of narrow therapeutic index
	Ruxolitinib	PD	heart rate (PR interval)	+++	antiarrhythmics, β-blockers, non-dihydropyridine calcium channel blockers, digitalis glycosides, cholinesterase inhibitors, sphingosine-1 phosphate receptor modulators, and HIV protease inhibitors
		PK	CYP1A2, CYP2B6, CYP2C9, CYP3A4	++	clarithromycin, erythromycin, diltiazem, ranolazine, itraconazole, ketoconazole, ritonavir, verapamil, grapefruit

Table S3. Drug-Drug interactions between emerged treatments for COVID-19 and respiratory medications (Clinical Significance: Serious–Use alternative= +++, Use with caution-Monitor= ++, moderate-minor=+)

Respiratory medications (ATC codes)		Interacting medications for COVID-19	Pharmacological Mechanism, Clinical outcome	Clinical Significance
**R03AC Selective β-2-adrenoreceptor agonists**	Salbutamol<br>Terbutaline<br>Fenoterol<br>Rimiterol<br>Hexoprenaline<br>Isoetarine<br>Pirbuterol<br>Tretoquinol<br>Carbuterol<br>Tulobuterol<br>Salmeterol<br>Formoterol<br>Clenbuterol<br>Reproterol<br>Procaterol<br>Bitolterol<br>Indacaterol<br>Olodaterol	Azithromycin, chloroquine, hydroxychloroquine, lopinavir	PD,<br>hERG -> QT-prolongation	++
		Dexamethasone, hydrocortisone, methylprednisolone	PD, Hypokalemia	+
**R03BA Glucocorticoids**	Budesonide,<br>Flunisolide,<br>Betamethasone,<br>Fluticasone,<br>Triamcinolone<br>Mometasone<br>Ciclesonide	atazanavir, ritonavir,	PK, CYP3A4 elevated drug concentrations	++
		anakinra, baricitinib, canakinumab	PD, myelossuppression, neutropenia	++
**R03BB Anticholinergics**	Revefenacin	atazanavir, lopinavir/ritonavir	PK, OAT1B1/ 1B3 elevated drug concentrations	++
**R03DA Xanthines**	Aminophylline,<br>Theophylline	azithromycin, ritonavir, anakinra, canakinumab, sarilumab, tocilizumab	PK, CYP3A4 and CYP1A2 abundance	++
		dexamethasone, hydrocortisone, methylprednisolone	PD, Hypokalemia	+
**R03DC Leukotriene receptor antagonists**	zafirlukast	chloroquine, hydroxychloroquine, remdesivir, colchicine, tocilizumab	PD Neuropathy & hepatotoxicity (remdesivir)	++
	zileuton	atazanavir, lopinavir/ritonavir, ruxolitinib	PK, CYP3A4	++
	montelukast	Dexamethasone, hydrocortisone, methylprednisolone	PK, CYP induction decrease drug concentration	+

**R06AD Phenothiazine derivatives**	Thiethylperazine, Methdilazine	ribavirin	CYP2D6	++
**R06AE Piperazine derivatives**	Cyclizine Chlorcyclizine Cetirizine Levocetirizine	ribavirin	Renal elimination	++
**R06AX Other antihistamines for systemic use**	Astemizole, Terfenadine	atazanavir, ribavirin	CYP3A4 elevated drug concentrations	++
		azithromycin, chloroquine, hydroxychloroquine	hERG -> QT-prolongation	+++
